# Characteristics of Invasive Fungal Infections among HIV Individuals from an Indigenous Origin in Mexico

**DOI:** 10.3390/jof4030109

**Published:** 2018-09-09

**Authors:** Mercedes Aranda-Audelo, Norma E. Rivera-Martínez, Dora E. Corzo-León

**Affiliations:** 1 Infectious Diseases Department, Hospital Regional de Alta Especialidad de Oaxaca, Avenida Aldama S/N, Centro, San Bartolo Coyotepec 71256, Oax, Mexico; mercedes.audelo@gmail.com (M.A.-A.); normaerm@gmail.com (N.E.R.-M.); 2 Medical Research Centre for Medical Mycology, Wellcome Trust Strategic Award, Institute of Medical Science, University of Aberdeen, King’s College, Aberdeen AB24 3FX, UK

**Keywords:** invasive fungal infections, histoplasmosis, IRIS, HIV, leukaemia, neutropenic fever, Mexico

## Abstract

In individuals with HIV/AIDS, 47% of the deaths are attributed to invasive fungal infections (IFIs), despite antiretroviral (ARV) therapy. This is a retrospective study carried out in the Hospital Regional de Alta Especialidad Oaxaca (HRAEO), southwest Mexico, where IFIs that occurred during 2016–2017 are described. A total of 55 individuals were included. Histoplasmosis (36%) and possible-IFIs in neutropenic fever (20%) were the most frequent cases, followed by cryptococcosis (14%). The HIV/AIDS subpopulation corresponded with 26 cases (47%), all from an indigenous origin. The incidence of IFIs among them was 24% (95% CI = 15–33%). The CD4+ T cells median was 35 cells/mL (IQR 12–58). Four cases (15%) of unmasking IRIS were identified, three of histoplasmosis and one coccidioidomycosis. Co-infections were found in 52% (12/23), and tuberculosis in 50% (6/12) was the most frequent. The mortality rate was 48%. The general characteristics of the HIV individuals who died were atypical pneumonia (70% vs. 9%, *p* = 0.01), acute kidney injury, (70% vs. 9%, *p* = 0.008) and ICU stay (80% vs. 9%, *p* = 0.002). In conclusion, IFIs are diagnosed in one out of four individuals with HIV/AIDS along with other complicated infectious conditions, leading to major complications and a high mortality rate.

## 1. Introduction

The incidence of invasive fungal infections (IFIs) has increased in the last few decades, particularly among individuals with conditions such as leukaemia or other malignancies, HIV infection, autoimmune diseases requiring long-term steroids or neutropenia secondary to toxic agents [1,2,3,4,5]. IFIs, similar to multidrug-resistant bacterial infections, have high mortality rates ranging between 40% and 80% [6,7,8]. Previous estimates have shown that IFIs cause as many deaths as tuberculosis and malaria [8,9]. In individuals with HIV/AIDS, although antiretroviral (ARV) therapy has dramatically improved the outcome of the HIV epidemic, 47% of AIDS-related deaths are attributed to IFIs [10]. This high rate of mortality associated with IFIs in the AIDS/HIV population can be reduced if efforts are focused on IFIs by improving access to diagnostic tools and to effective drugs [10,11]. 

*Pneumocystis* infections, cryptococcal meningitis and disseminated histoplasmosis are considered the most life-threatening IFIs in HIV/AIDS populations and, as a group, one of the ten most frequent serious fungal infections in Mexico after invasive candidiasis [12].

In this study, we report an analysis of the clinical characteristics found in IFIs among HIV/AIDS individuals in a Mexican indigenous population.

Our study focused on an HIV population of indigenous origin in southwest Mexico, due to the poor information available about infectious complications related to HIV in these populations and the null evidence specifically about IFIs. Attending to these individuals on a daily basis allowed us to describe the epidemiology and characteristics of IFIs among them, as well as the differences in the mortality rates and factors associated with death.

## 2. Materials and Methods

### 2.1. Study Design and Data Collection

During 2015, in Oaxaca, 3,976,297 inhabitants were registered; 53% of them lived in rural areas; the population was mainly younger people (median 26 years); and 66% of the population was considered to be indigenous [13].

The Hospital Regional de Alta Especialidad Oaxaca (HRAEO) is the only tertiary care referral hospital located in the state of Oaxaca, 550 km southwest from Mexico City. This hospital is a referral centre mainly for individuals having onco-haematological conditions, primary and secondary immunocompromised and seriously ill patients. The hospital capacity is 66 hospital beds, 10 ICU beds and 13 emergency department beds. 

This is a retrospective and transversal study carried out in the Hospital Regional de Alta Especialidad Oaxaca (HRAEO). This study was conducted in accordance with the Declaration of Helsinki and approved on 29 June 2017 by the local IRB with the following register number, HRAEO-CIC 003-17.

IFIs that occurred in the HRAEO during 2016 and 2017 were identified using the hospital database, which classifies diseases according to the International Statistical Classification of Diseases and Related Health Problems Version 10 (ICD-10). Clinical data were recovered from medical records using a collection data sheet. Data collected included demographics, previous comorbidities, chief complaint, type of IFI, site of isolation, diagnostic tools, antiretroviral therapy status, viral load and CD4+ levels. The type and duration of antifungal therapy, as well as the development of acute kidney injury (AKI) and liver damage (LD) during antifungal therapy were documented. AKI was defined as an increase of ≥2-times in serum creatinine levels compared with the baseline level and/or the presence of hypokalaemia or hypomagnesemia during antifungal therapy. Meanwhile, LD was defined as an increase in the ratio aspartate transaminase (AST)/alanine transaminase (ALT) or total bilirubin ≥2-times compared with the baseline level during antifungal therapy. Outcomes of interest recorded were mortality, stay at ICU, length of stay (LOS) in hospital and ICU. 

The mycological diagnosis of IFIs was done in the HRAEO clinical microbiology laboratory and the Institute of Medical Sciences reference clinical microbiology laboratory in Mexico City using culture and non-culture techniques. Clinical specimens from blood, spinal fluid and bronchioalveolar lavage were cultured in Sabouraud dextrose agar with cycloheximide and the Bact-Alert 3D microbiological identification system (bioMérieux, Marcy l’Etoile, France). The identification of species was done by macroscopic appearance and microscopic identification using lactophenol cotton blue staining. Finally, yeast and yeast-like organisms were identified and tested for antifungal susceptibility using Vitek 2 XL (bioMérieux, France). *Coccidioides* Antibody Latex Agglutination (IMMY, Norman, OK, USA), the Cryptococcal Antigen Latex Agglutination System (Meridian Bioscience, Cincinnati, OH, USA) and leukocyte concentrate from peripheral blood were the non-culture techniques used in the diagnosis of IFIs. All biopsies were evaluated by the histology department with Wright, silver-methenamine and periodic acid staining.

IFIs were classified according the European Organisation for Research and Treatment of Cancer/Invasive Fungal Infections Cooperative Group and the National Institute of Allergy and Infectious Diseases Mycoses Study Group (EORTC/MSG) criteria [5]; however, we also, included a definition of possible/suspected disseminated *Histoplasma* infections for the HIV population, which is not included in the classical EORTC/MSG criteria. These possible/suspected infections were defined as in previous reports [14]; briefly, those HIV patients with fever, weight loss, dyspnoea, diffuse localised radiological infiltrates, usually in a miliary pattern or as atypical pneumonia, with or without lymph node involvement and/or hepatosplenomegaly and with or without pancytopenia. We added to this clinical definition the null response to anti-tuberculosis therapy or when tuberculosis was previously ruled-out and when the patient responded to antifungal therapy and no evidence of other fungal infection existed. Proven and probable IFIs received diagnosis-driven therapy, meanwhile, possible IFIs received empiric therapy. The incidence rate of IFIs in the HIV population was estimated based on the number of HIV patients attended to during the study period and the newly-diagnosed IFIs. Cases of probable immune response inflammatory syndrome (IRIS) were identified. Probable unmasking IRIS was defined as the diagnosis of a new opportunistic infection (OI), an IFI in this case, with a pronounced inflammatory component within the first eight weeks after initiation or a change in ARV [15].

Once the IFIs were identified, we determined a person to have an indigenous origin if, in the medical charts, one of the following characteristics were indicated: the person considers himself/herself to be from an indigenous origin, speaks a known indigenous language and lives within an indigenous community. If this information was not available in the medical records, we did not count that person as having an indigenous origin.

### 2.2. Statistical Analysis

Data are presented as a proportion, median and interquartile range (IQR) depending on the type of data. For categorical data comparisons, Pearson’s or Fisher’s tests were used as required, and for ordinal and quantitative values, the Mann–Whitney test was used. Statistical analysis and plot construction were done using SPSS 24 (IBM, Chicago, IL, USA) and Prism 7 (Graph Pad, La Jolla, CA, USA) software.

## 3. Results

### 3.1. General Characteristics of All the IFIs Found during the Study Period

A total of 55 individuals with IFIs were diagnosed in the HRAEO during 2016–2017 and were included in this report. Forty-eight individuals had an indigenous origin (87%), and the indigenous origin could not be determined retrospectively in seven individuals. At the beginning of the antifungal therapy, most of the IFIs were classified as proven (69%) and 31% as possible IFIs. Two cases had microbiological evidence of IFI after beginning antifungal therapy, in one case during the therapy and the other case post-mortem. Blood, lung secretion and spinal fluid cultures provided the diagnosis of 77% (31/40) of the proven IFIs; see Table 1. 

Histoplasmosis and possible IFIs in neutropenic patients were the most frequent IFIs (36% and 20%, respectively), followed by cryptococcosis (14%) and candidemia (13%), among others; see Table 1. Median hospital length of stay (LOS) was 20 days (IQR = 11–38); an ICU stay was required in 31% of cases for a median of four days (IQR = 3–8); and the mortality rate was 43%; see Table 1. 

Fluconazole and amphotericin were the two drugs of choice as antifungal therapy; see Table 2. The median duration of antifungal therapy was 25 days (IQR 10–63), and AKI and LD were diagnosed in 31% and 20% of the cases, respectively, during the antifungal therapy. 

### 3.2. Characteristics of the IFIs in the HIV Population and Factors Associated with Mortality 

**Figure 1:**
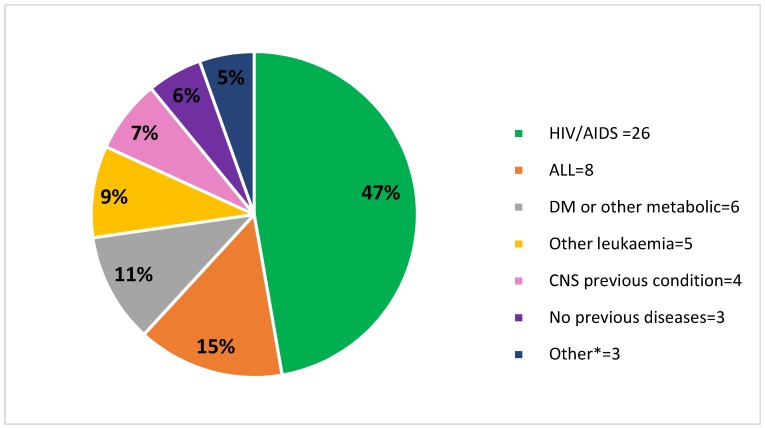
HIV infection was the predominant comorbidity found in the population with IFIs. ALL: acute lymphocytic leukaemia, CNS: central nervous system, DM: diabetes mellitus. * Other: liver disease, chronic obstructive pulmonary disease.

HIV infection was the predominant comorbidity (26/55, 47%) in our study population, followed by leukaemia (13/55, 24%) and diabetes (6/55, 11%); see Figure 1. The indigenous origin was documented for all the individuals in the HIV population. 

During 2016 and 2017, fungal infections were diagnosed in 13 out of 48 (27%) and 13 out of 60 (22%) hospitalised individuals with HIV, respectively. Therefore, the incidence of IFIs among in-hospital individuals with HIV was 24% (95% CI = 15–33%). 

Individuals with HIV and fungal infections were predominantly male (77%) and younger (median 33 years, IQR 28–43); see Table 1. The CD4+ T cell median count was 35 cells/mL (IQR 12–58), and only four patients had CD4+ T cell counts >100 (three of them had disseminated histoplasmosis and one cryptococcal meningitis). Viral load (VL) median count was 99 × 10^3^ (13–203 × 10^3^), and only five persons had undetectable VL. Four cases (4/26, 15%) of unmasked IRIS were identified between four and eight weeks after starting ARV. IRIS due to IFIs were three cases of histoplasmosis and one case of coccidioidomycosis. Two out of four probable IRIS died, and both were due to histoplasmosis.

Co-infections with IFIs were found in 52% (12/23) of the cases, with tuberculosis (50%, 6/12) and bacterial infections (33%, 4/12) being the most frequent non-fungal associated co-infections. The most important chief complaint in HIV populations with IFIs was neurological syndrome (9/26, 35%). This neurological syndrome could have been presented as seizures, meningitis or palsy. The second more frequent chief complaint was atypical pneumonia, with or without pancytopenia (8/26, 31%); see Table 1. Most of the atypical pneumonia cases had the following radiologically features: diffuse bilateral micronodular pattern (5/8), interstitial infiltrates (4/8) and (2/8) tree-in-bud sign, (1/8) with spontaneous pneumothorax.

The most frequent fungal infection in the HIV population was histoplasmosis (16/26, 61%), followed by cryptococcosis (7/26, 27%); see Table 1. The majority of IFIs, in the HIV group, were diagnosed by culture, 22/26 (85%), mainly blood culture and spinal fluid (15/26, 58%); serologic diagnosis was made only for one case of coccidioidomycosis; and cryptococcal antigen in spinal fluid was positive in all the cases with meningitis; see Table 3. 

The general characteristics of the HIV individuals who died were atypical pneumonia (70% vs. 9%, *p* = 0.01), amphotericin B deoxycholate (AMBD) was not used as the first antifungal (10% vs. 64%), acute kidney injury, (70% vs. 9%, *p* = 0.008) and ICU stay (80% vs. 9%, *p* = 0.002); see Table 4.

### 3.3. Histoplasmosis among HIV/AIDS Individuals 

During the study period, the incidence rate of histoplasmosis in hospitalised HIV individuals was 15% (95% CI = 9–23%). Proven histoplasmosis accounted for 75% of cases (12/16). Four cases were classified as possible or suspected histoplasmosis with good response to antifungal therapy; see Table 3. The clinical characteristics of proven and possible histoplasmosis cases were not significantly different.

Disseminated histoplasmosis was the most frequent clinical presentation (15/16, 94%). The median CD4+ T cell count among individuals with histoplasmosis was 33 cells/mL (IQR 10–59), lower than HIV individuals with a different IFI, but not statistically significant (38 cells/mL, IQR 10–59, *p* = 0.84). Histoplasmosis was associated with another infection in 63% of cases (10/16). The proportion of identified co-infections was higher for the group with histoplasmosis (63%) when compared with other IFIs in this studied HIV population 17% (2/8), but was not significantly different (*p* = 0.09). The most frequent co-infection was tuberculosis (60%, 6/10 co-infections), followed by bacterial infection (30%, 3/10); see Table 5. All cases of co-infection with tuberculosis occurred in individuals with histoplasmosis. 

## 4. Discussion

The study of the HIV/AIDS pandemic over the years has improved and included neglected populations. Despite this improvement, indigenous populations remain neglected due to several reasons. Some reasons are the lack of health programs considering their language, ideologies and traditions [16,17]. Furthermore, other related factors are social stereotypes, discrimination and the misconception that HIV will not affect these populations because their communities are assumed to be heterosexual and monogamic [16,17]. In Mexico, as with the rest of Latin America, the risk of contracting the infection in indigenous and rural populations is strongly associated with migration patterns [18,19,20]. Examples of infections associated with migration are the cases of coccidioidomycosis found in our study; coccidioidomycosis is endemic in states in the Mexican northwest, not in the southwest of Mexico [12]. The HIV epidemic has increased in indigenous populations, with prevalence reaching 20-times higher than non-indigenous populations, and the risk of death is at least three-times higher due to HIV-related complications [16,17,20]. Considering that indigenous individuals had a higher prevalence of HIV in previous studies, we hypothesised that IFIs could also be as frequent and deadly in these individuals. Our findings showed that one in four individuals with HIV presented an IFI in this population, and half of these infections were fatal.

Outcomes of IFIs, ICU stay, AKI and mortality were similar to previous reports, where mortality due to histoplasmosis and cryptococcosis ranged between 6% and 35% [21,22,23]. In these previous reports, AKI and fungaemia were shown to be factors associated with higher mortality rates in cryptococcosis and histoplasmosis, independent of the HIV status [21,23]. In our study, we found AKI and ICU stay more frequently in individuals who died. The association of ICU stay and AKI with higher mortality could be the result of several related factors, such as higher severity of infections, dissemination and fungaemia.

Non-culture diagnostic techniques are not always available in referral centres in Mexico and in rural areas, except for the cryptococcal antigen. Lacking these tests leads to the use of empiric antifungal therapy based on possible cases of histoplasmosis and neutropenic fever in the current study population. The definition of possible histoplasmosis is not considered within the EORTC criteria [5], but has previously been used [24] due to the necessity of identification of cases in the context of no other resource. The possible cases of histoplasmosis identified in this study were neither statistically or clinically different from proven cases; however, more studies determining the accuracy of this definition are necessary. The use of this definition as a surrogate for non-culture diagnostic techniques does not replace them. These techniques would improve the diagnostic capacity of hospitals attending rural populations. Therefore, we would consider it compulsory to advocate for rural populations by highlighting the necessity of having these diagnostic tests available, via publications such as this one.

We also recognise that there are improvements in the health care and clinical approach these infections require. One of them is the enhancement of the local diagnostic capacity with cultures and cytology testing [25]. In our study, the high frequency of IFIs, specifically histoplasmosis, in co-infection with tuberculosis in HIV/AIDS individuals, makes necessary a standardised clinical approach for similar clinical contexts. These findings were also previously reported in other Latin American populations [24,26,27,28]. Hence, we highly recommend, performing blood and bone marrow cultures, peripheral and bone marrow blood smears, along with hepatic enzymes and LDH, even though tuberculosis has been already diagnosed, in the context of an HIV individual with <50 CD4+ cells and pancytopenia with or without atypical pneumonia. 

In 2015, an estimation of the burden of serious fungal infections in Mexico reported histoplasmosis to be probably the third most important IFI in HIV populations, after *Pneumocystis* infections and cryptococcosis [12]. The estimation, performed in the mentioned study, was made using incidence rates from the early 1990s and considered the burden to be underestimated, as no updates from continuous surveillance programs have been done in Mexico since then. The current study contributes to a better panorama of the state of these IFIs in Mexico. 

This study showed that histoplasmosis was the most important IFI in the HIV population studied, with higher frequency than cryptococcosis; this finding could be partly explained by the endemic area, for this infection, where Oaxaca is localised [29]. Similar trends have been previously reported for similar HIV/AIDS populations in western French Guiana [24] and Guatemala [28]. Compared with the French Guiana population and the Guatemalan report, our population showed a higher proportion of co-infection between histoplasmosis and tuberculosis. This finding requires further investigation to explain how host and microbial factors could be interacting. 

The incidence of unmasked IRIS for all causes has been reported previously to be 5% (CI 95% = 3.3–7.3%) in an HIV/AIDS population in South Africa [30]. In our study, this proportion of IRIS, only for IFIs, was twice as high. Furthermore, interestingly, the type of IFIs associated with reconstitution syndrome was different from other reports. Cryptococcosis is usually the most frequently reported IFI in unmasking IRIS [30,31]; on the contrary, in our study, it was histoplasmosis and coccidioidomycosis. This finding may be a result of the endemic area where the study was carried out, deep immunosuppression in our population and also the size of the sample. Further follow-up to this phenomenon would be required. This study has several limitations due to its retrospective design and small sample; however, it gives an important panorama on the impact and frequency of IFIs in the HIV/AIDS populations of indigenous origin. 

## 5. Conclusions

IFIs are diagnosed in one out of four individuals with HIV/AIDS, along with other complicated infectious conditions such as tuberculosis, leading to major complications and high mortality rates.

## Figures and Tables

**Table 1:** General characteristics of the studied population. IFI, invasive fungal infection.

Characteristic	Total *n* = 55 (%)	HIV *n* = 26 (%)
Gender		
Female	20 (36)	6 (23)
Age (median, IQR)	34, 27–41	33, 28–43
Year of diagnosis		
2016	35 (64)	13 (50)
2017	20 (36)	13 (50)
Chief complaint		
Seizures, meningitis or palsy	11 (20)	9 (35)
Neutropenic fever	9 (16)	0
Atypical pneumonia with pancytopenia	7 (13)	6 (23)
Atypical pneumonia	5 (9)	2 (8)
Pancytopenia	5 (9)	2 (8)
Lymphadenopathies with or without fever	5 (9)	4 (15)
Another pulmonary syndrome	4 (7)	1 (4)
Cutaneous dermatosis or nodules	2 (4)	2(8)
Miscellaneous *	7 (13)	0
Indication of antifungal therapy		
Proven IFI (diagnosis driven therapy)	38 (69)	21 (81)
Possible IFI (empiric therapy)	17 (31)	5 (19)
Type of fungal infection or syndrome indicating antifungal therapy		
Histoplasmosis	20 (36)	16 (61)
Neutropenic fever	11 (20)	0
Cryptococcosis	8 (14)	7 (27)
Candidemia	7 (13)	0
Coccidioidomycosis	3 (5)	1 (4)
Combined IFI *	3 (5)	2 (8)
Mucormycosis	2 (4)	0
Aspergillosis	1 (2)	0
Site of the isolation/identification		
Blood	17 (31)	8 (31)
Lung (BAL, endotracheal culture)	8 (14)	2 (8)
CNS/spinal fluid	6 (11)	5 (19)
Lymph node	4 (7)	3 (11)
Skin	3 (5)	2 (8)
Blood and CNS/spinal fluid	2 (4)	2 (8)
No microbiological evidence of IFI	15 (27)	4 (14)
ICU stay	17 (31)	9 (35)
Length of stay at hospital	20, 11–38	16, 7–36
Length of stay at ICU	4, 3–8	3, 2–5
Survived (*n* = 47)	27 (57)	11 (52)

* Miscellaneous: burns, surgical complications, diabetic foot, liver injury. combined IFI: candidemia + possible histoplasmosis (1), candidemia + *Trichosporon asahii* infection (1), fusariosis + candidemia (1), CNS: central nervous system. BAL: bronchoalveolar lavage.

**Table 2:** Characteristics of antifungal therapy. AKI, acute kidney injury. AMBD, amphotericin B deoxycholate.

Characteristic	Total *n* = 52 (%) *	HIV *n* = 26 (%)
First antifungal used		
Fluconazole	21 (38)	9 (35)
Amphotericin B deoxycholate	16 (29)	9 (35)
Itraconazole	11 (20)	8 (31)
Caspofungin	4 (7)	0
Second antifungal used *		
Fluconazole	7 (27)	4 (25)
AMBD	7 (27)	5 (31)
Itraconazole	5 (19)	5 (31)
Voriconazole	4 (15)	1 (6)
Caspofungin	2 (8)	0
LAMB	1 (4)	0
Duration of antifungal 1	10, 5–15	9, 5–15
Duration of antifungal 2	13, 3–39	20, 5–50
AKI during antifungal therapy	16 (31)	9 (35)
Liver damage during antifungal therapy *n* = 50	10 (20)	4 (17)

* 52/55 (94%) individuals received an antifungal drug; 26/52 (50%) individuals required a different antifungal (10/26 HIV individuals and 16/29 non-HIV individuals).

**Table 3:** Diagnostic tools used for the diagnostic approach of IFIs in HIV individuals of indigenous origin.

Type of Fungal Infection	Clinical Syndrome	Diagnostic Tool
Histoplasmosis	Disseminated *n* = 15	Clinical suspicion * plus radiological findings plus not responding to other therapies, improving with antifungal ** (*n* = 4)
		Lymph node biopsy, culture (*n* = 2/3) and histopathology (*n* = 1/3)
		Skin biopsy (*n* = 1)
		Respiratory secretion culture (*n* = 1)
		Bone marrow culture (*n* = 3/5), blood culture (*n* = 3/5), peripheral blood smear (*n* = 1/5)
	Cutaneous histoplasmosis *n* = 1	Skin biopsy (*n* = 1)
Cryptococcosis	Meningitis *n* = 4	Spinal fluid culture (*n* = 4/4), antigen (*n* = 4/4)
	Two sites affected *n* = 3	Spinal fluid culture (*n* = 1/3), spinal fluid plus blood culture (*n* = 2/3)
Coccidioidomycosis	Pulmonary (*n* = 1)	Serologic diagnosis (*n* = 1)
Fungal co-infection	*Trichosporon asahii* and candidemia (*n* = 1)	Blood culture
	Histoplasmosis and candidemia (*n* = 1)	Blood culture

* Clinical suspicion: miliary or micronodular patterns on the chest imaging test, adenopathies and hepatosplenomegaly. ** Other therapies used: anti-tuberculosis treatment, antibacterial management.

**Table 4:** Characteristics of dead and survivor individuals in the HIV population of indigenous origin. Univariate analysis.

Characteristic *	Died *n* = 10 (%)	Survived *n* = 11 (%)	*p*-Value
Gender (Female)	1 (10)	4 (36)	0.31
Age, years (median, IQR)	36, 28–51	32, 27–40	0.70
CD4+ T cells count, cells/mL, (median, IQR)	32, 16–39	51, 12–179	0.36
Viral load, cells/mL, (median, IQR)	105 × 103, 29–207 × 103	143 × 103, 0–145 × 103	0.83
Co-infection other than fungi (18/21, 86%)	5 (62)	6 (60)	1
Clinical syndrome associated with IFI			
Atypical pneumonia	7 (70)	1 (9)	0.01
CNS/PNS involvement	2 (20)	4 (36)	
Other different	1 (10)	6 (55)	
Type of IFI			
Proven IFI	8 (80)	8 (73)	1
Probable IFI	2 (20)	3 (27)	
Specific IFI			
Histoplasma	7 (70)	8 (73)	0.22
Cryptococcosis	1 (10)	3 (27)	
Combined IFI **	2 (20)	0	
Site of isolation			
Blood	7 (70)	3 (27)	0.05
Other than blood	3 (30)	8 (73)	
First antifungal administrated			
Fluconazole	3 (30)	2 (18)	0.04
Itraconazole	6 (60)	2 (18)	
AMBD ***	1 (10)	7 (64)	
Second antifungal administrated			
Fluconazole	1 (33)	2 (22)	0.29
Itraconazole	0	4 (44)	
AMBD ***	2 (67)	1 (11)	
LAMB	0	1 (11)	
Voriconazole	0	1 (11)	
Acute kidney injury	7 (70)	1 (9)	0.008
Liver damage	4 (40)	0	0.09
ICU *	8 (80)	1 (9)	0.002
Receiving ARV therapy at the diagnosis of IFI			
NO	4 (40)	3 (27)	0.69
YES	6 (60)	8 (73)	

* Data of 21/26 patients were available; ** combined IFIs: candidemia + possible histoplasmosis (1), candidemia + *Trichosporon asahii* infection (1); *** during 2016 and part of 2017, AMBD was not available in the country due to problems of distribution and production. AMBD: amphotericin B deoxycholate, LAMB: liposomal amphotericin.

**Table 5:** Clinical context of histoplasmosis in the HIV/AIDS population of indigenous origin.

Characteristic	*n* = 16
Clinical syndrome	
Disseminated	15 (94)
Cutaneous	1 (6)
CD4+ T, cells/mL, (median, IQR)	33, 10–59
Co-infection with tuberculosis	6 (37)
Diagnostic tools	
Blood culture or bone marrow culture	6 (33)
Lymph node biopsy/culture	3 (16)
Skin biopsy/culture	2 (11)
Bronchoalveolar lavage	1 (5)
Clinical syndrome, no microbiological evidence, imaging, but response to antifungal therapy	4 (22)
